# First impressions: A prospective evaluation of patient–physician concordance and satisfaction following the initial medical oncology consultation

**DOI:** 10.1002/cam4.6758

**Published:** 2023-12-08

**Authors:** Yvonne Bach, Elan Panov, Osvaldo Espin‐Garcia, Eric Chen, Monika Krzyzanowska, Grainne O'Kane, Malcolm Moore, Rebecca M. Prince, Jennifer Knox, Robert Grant, Lucy X. Ma, Michael J. Allen, Lawson Eng, Ekaterina Kosyachkova, Thais Baccili Cury Megid, Carly Barron, Xin Wang, Marie‐Philippe Saltiel, Abdul Rehman Rehman Farooq, Raymond W. Jang, Elena Elimova

**Affiliations:** ^1^ Princess Margaret Cancer Centre Toronto Ontario Canada

**Keywords:** Gastrointestinal neoplasms, medical oncology, psycho‐oncology, patient communication, prognosis

## Abstract

**Background:**

An especially significant event in the patient–oncologist relationship is the initial consultation, where many complex topics—diagnosis, treatment intent, and often, prognosis—are discussed in a relatively short period of time. This study aimed to measure patients' understanding of the information discussed during their *first* medical oncology visit and their satisfaction with the communication from medical oncologists.

**Methods:**

Between January and August 2021, patients without prior systemic treatment of their gastrointestinal malignancy (GI) attending the Princess Margaret Cancer Centre (PMCC) were approached within 24 h of their initial consultation to complete a paper‐based questionnaire assessing understanding of their disease (diagnosis, treatment plan/intent, and prognosis) and satisfaction with the consultation. Medical oncology physicians simultaneously completed a similar questionnaire about the information discussed at the initial visit. Matched patient–physician responses were compared to assess the degree of concordance.

**Results:**

A total of 184 matched patient–physician surveys were completed. The concordance rates for understanding of diagnosis, treatment plan, treatment intent, and prognosis were 92.9%, 59.2%, 66.8%, and 59.8%, respectively. After adjusting for patient and physician variables, patients who reported treatment intent to be unclear at the time of the consultation were independently associated with lower satisfaction scores (global *p* = 0.014). There was no statistically significant association between patient satisfaction and whether prognosis was disclosed (*p* = 0.08).

**Conclusion:**

An in‐depth conversation as to what treatment intent and prognosis means is reasonable during the initial medical oncology consultation to ensure patients and caregivers have a better understanding about their cancer.

## Graphical abstract

This study aimed to measure gastrointestinal cancer patients' understanding of the information discussed and their satisfaction with the communication during their first medical oncology visit. We found that patient–physician concordance rates were low in treatment intent (palliative vs. curative intent) and prognosis.

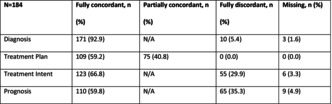

## 1 INTRODUCTION

Gastrointestinal (GI) malignancies account for 26% of all new cancer cases and 35% of all cancer‐related deaths globally.^1^ Systemic chemotherapy is an integral component of the multidisciplinary treatment of GI cancers and most patients are seen by a medical oncologist at some point in their cancer journey in addition to assessments by surgical and/or radiation oncologists. Due to the complex nature of the disease and the involvement of multiple specialties, it is critical that patients and families understand their cancer diagnosis, the purpose of each treatment modality, and prognosis, so that they can make informed decisions regarding their care.

In previous studies that measured comprehension and satisfaction with the information given to newly diagnosed lung cancer patients, concordance between patients and physicians were highest in diagnosis (86%–90%) and type of treatment (81%–83%) but relatively low in treatment intent (42%–49%).^2^, ^3^ The agreement between patients and physicians on cancer curability in other studies ranged between 29% and 76%, although patient cohorts were variable in disease site and stage in cancer journey.^4^, ^5^, ^6^, ^7^ Furthermore, physician cohorts were heterogeneous with respect to medical specialty and level of experience dealing with serious illnesses.^2^, ^3^, ^4^, ^5^, ^6^, ^7^

Prognostic disclosure following a cancer diagnosis has been a more difficult topic for physicians to address across all parts of the world.^8^, ^9^, ^10^, ^11^, ^12^, ^13^, ^14^, ^15^, ^16^ In a multicenter study, over 70% of patients diagnosed with metastatic solid malignancies in the United States wanted to be told their life expectancy.^10^ However, only 17.6% recalled a prognosis being disclosed by their oncologists. This discordance is concerning, as an accurate understanding of prognosis may help patients and caregivers with the decision‐making process and manage their expectations as they begin treatment. Effective communication skills and patient‐centered care have been key focus areas in improving health literacy, decision‐making, and clinical outcomes in cancer patients.^17^, ^18^, ^19^, ^20^, ^21^, ^22^, ^23^ In fact, many consultation aids have been developed for both physicians and patients to optimize resources and improve communication at the initial visit in an oncology setting.^24^, ^25^, ^26^, ^27^, ^28^, ^29^, ^30^, ^31^, ^32^, ^33^

In this study, we aimed to evaluate the effectiveness of physician‐patient communication during a patient's *first* medical oncology consultation at the Gastrointestinal Oncology Clinic at the Princess Margaret Cancer Centre (PMCC), a tertiary referral center in Toronto, Canada, to identify key areas in which we can improve clinical care based on the needs and values of our patients and caregivers. We chose to focus on the first consultation as it is often an emotionally overwhelming and an information‐heavy visit. We also wanted to assess a more homogenous group of physicians such as medical oncologists, who would be more adept in delivering the type of sensitive news specific to cancer patients. The primary objective of this study was to quantify patient–physician concordance in understanding of diagnosis, treatment modality and intent, and prognosis of GI malignancies. The secondary objective was to investigate whether patient or physician characteristics were associated with patient satisfaction on communication delivered at the initial consultation.

## 2 METHODS

### 2.1 Study design and population

This was a prospective study that recruited consecutive patients (≥18 years old) with a confirmed diagnosis of a GI malignancy (i.e., gastroesophageal, small intestinal, colorectal, anal canal, hepatic, pancreatic, and biliary cancers) during their first encounter with a medical oncologist at the PMCC. Medical oncologists who participated in this study all subspecialized in GI malignancies. Between January and August 2021, patients without prior systemic treatment for their GI malignancy were approached within 24 h of their initial consultation for voluntary participation in a paper‐based questionnaire assessing understanding of their disease and satisfaction with the communication surrounding their cancer.

Patients were excluded if they met one or more of the following criteria: (1) Non‐English speaking patients who were not accompanied by an English‐speaking caregiver at the consultation (as the study questionnaire was provided only in English); (2) patients already receiving chemotherapy or have received chemotherapy for their GI malignancy; (3) enrolled patients who were unable to complete the questionnaire by their second clinic visit; and (4) patients with poor performance status (i.e., Eastern Cooperative Oncology Group (ECOG) score 3–4) as judged by the patient's health‐care team.

### 2.2 Survey instrument and measures

Informed consent was obtained from each patient. Patients were required to complete the study questionnaire after their consultation in clinic or at home and return it to the study coordinator prior to the second clinic visit.

#### 2.2.1 Patient understanding

Patient understanding of the information delivered during the initial consultation was measured with a questionnaire adapted from previous patient recall studies^2^, ^3^ and input from the clinical team. The questionnaire composed of multiple choice and open‐ended questions on demographics (age, sex, educational level, primary language, and whether a family member or friend was present during the initial consultation) and the information discussed during the consultation (cancer diagnosis, treatment options, treatment intent [curative vs. palliative], and prognosis) (Data S1). Staff medical oncologists completed a similar version of the questionnaire for each patient who consented to the study. The physician questionnaire was also composed of open‐ended and multiple choice questions on demographics (years in practice, whether a medical trainee saw the patient first) and the information discussed during the consultation (Data S2). Patient and physician questionnaires were completed separately and responses were not shared with either group. The responses were then compared and the degree of congruence were defined as fully concordant, partially concordant, and fully discordant. Each category was scored either fully concordant or fully discordant, except for treatment plan, in which patients and physicians were allowed to select more than one option (e.g., chemotherapy *and* radiotherapy). A response was *partially* concordant if the patient and physician did not select all the same options but had at least one common answer selected.

#### 2.2.2 Patient satisfaction with communication

The satisfaction survey consisted of 10 statements adapted from Schofield et al.,^16^ which assessed how clearly diagnosis, treatment plan, treatment intent, and prognosis were communicated. A 5‐point Likert scale was employed (strongly disagree, disagree, neither agree or disagree, agree, or strongly agree). If all 10 statements were completed by the patient, a total satisfaction score was calculated for a maximum of 50 points (10 questions × 5 points/question). Total satisfaction scores were stratified by patient response to what information was given for treatment intent and prognosis (items 7 and 8 on Data S1, respectively).

### 2.3 Data collection and analysis

Study data were de‐identified, collected, and managed using REDCap electronic data capture tools hosted at the University Health Network.^34^ REDCap (Research Electronic Data Capture) is a secure, web‐based software platform designed to support data capture for research studies, providing (1) an intuitive interface for validated data capture; (2) audit trails for tracking data manipulation and export procedures; (3) automated export procedures for seamless data downloads to common statistical packages; and (4) procedures for data integration and interoperability with external sources.

Data were reported as means and standard deviations for continuous variables and as counts and percentages for categorical variables. A univariable logistic regression analysis was performed to assess patient‐ and physician‐based factors associated with concordance on treatment intent and prognosis. Patient‐based factors included age, sex, prior cancer history, educational level, primary language, whether the patient completed the study questionnaire at home, whether a family member or friend accompanied the patient, whether a patient has had a consultation with an external medical oncologist or surgical/radiation oncologist, and patient perception of treatment intent, and prognosis. Physician‐based factors included years of practice in medical oncology and whether the patient was first assessed by a medical trainee (fellow, resident, physician assistant). Similarly, a uni/multivariable linear regression analysis was conducted using the same variables on patient satisfaction levels. Statistically significant results were defined with *p* ≤0.05. Statistical analyses were performed using R v4.1.2.

## 3 RESULTS

A total of 184 matched patient–physician surveys were completed. The mean age of patients was 64.5 ± 12.3 years (Table 1). One fifth of patients had a prior history of a second primary cancer. Based on the oncologists' answers, there was a higher proportion of patients seen for palliative versus curative intent therapy (*n* = 85 [46.7%] vs. *n* = 58 [31.8%], respectively). In our patient group, 84 (45.6%) were female, 112 (60.9%) had a college degree or higher, and 143 (77.7%) selected English as their primary language. Thirty‐six (19.6%) patients had already seen an external medical oncologist and were referred for either a second opinion and/or potential participation in clinical trials. One hundred twenty‐three (66.8%) patients had also seen a radiation and/or surgical oncologist prior to their medical oncology consultation at PMCC. Nearly 75% (*n* = 137) of patients had a family member or friend that accompanied the patient at their consultation and was able to assist in completing the study questionnaire. Nine staff medical oncologists participated in the study. The mean number of years of experience as a staff medical oncologist was 13.1 ± 11.0 years (range: 2–34 years). More than half of the study patients (*n* = 115; 62.5%) were seen by a medical oncologist within 10 years of appointment.

**TABLE 1:** Patient demographics.

Variables	*N* = 184
Age, years (mean ± SD)	64.5 ± 12.3
Sex, female, *n* (%)	84 (45.6)
Treatment intent^a^	
Curative, *n* (%)	58 (31.8)
Palliative, *n* (%)	85 (46.7)
More investigations needed, *n* (%)	39 (21.4)
Not answered, *n*	2
Prior history of cancer (%)	37 (20.1)
Education level	
College level or above, *n* (%)	112 (68.3)
High school or below, *n* (%)	52 (31.7)
Not answered, *n*	20
Primary language	
English, *n* (%)	143 (79.4)
Other, *n* (%)	37 (20.6)
Not answered, *n*	4
Seen by other medical oncologist prior to PMCC consultation, *n* (%)	36 (19.6)
Seen by radiation or surgical oncologist prior to medical oncologist consultation at PMCC, *n* (%)	123 (66.8)
Family member present at PMCC consultation, *n* (%)	137 (74.5)
Patients seen by a physician with ≤10 years of medical oncology experience, *n* (%)	115 (62.5)
Years of experience as a staff medical oncologist^a^	
Mean ± SD	13.1 ± 11.0
Median (min, max)	9 (2,34)
Patients seen by a medical trainee before staff oncologist, *n* (%)	141 (76.6)

Abbreviation: PMCC, Princess Margaret Cancer Centre.

^a^ Based on physicians' answer on questionnaire.

### 3.1 Patient–physician concordance

Patient–physician concordance was divided into four categories: (1) diagnosis, (2) treatment plan, (3) treatment intent, and (4) prognosis (Table 2). More than 90% (*n* = 171) of patients agreed with their medical oncologist on the cancer disease site and 100% (*n* = 184) of patients were either fully (*n* = 107; 59.2%) or partially (*n* = 75; 40.8%) concordant on their understanding of their treatment plan. The concordance rate for treatment intent was 66.8% (*n* = 123). Of the 55 patients whose response for treatment intent was discordant from the physician's, 42 (76.4%) patients believed the intent of treatment to be curative or unclear when in fact the physician reported them to be palliative.

**TABLE 2:** Patient–physician concordance rates on understanding of cancer diagnosis, treatment plan, treatment intent, and prognosis.

*N* = 184	Fully concordant, *n* (%)	Partially concordant, *n* (%)	Fully discordant, *n* (%)	Missing, *n* (%)
Diagnosis	171 (92.9)	N/A	10 (5.4)	3 (1.6)
Treatment plan	109 (59.2)	75 (40.8)	0 (0.0)	0 (0.0)
Treatment intent	123 (66.8)	N/A	55 (29.9)	6 (3.3)
Prognosis	110 (59.8)	N/A	65 (35.3)	9 (4.9)

The concordance rate for prognosis was 59.8% (*n* = 110). Of the 65 discordant cases, 22 (33.8%) patients reported that prognosis was not discussed with them when physicians reported otherwise and six (9.2%) patients overestimated their prognosis to ≥1 year when physicians reported <1 year. Based on physician‐reported responses, the topic of prognosis was not addressed with 113 out of 184 (61.4%) patients, with an additional five (2.7%) patients requesting not to discuss it. Of the patients with whom prognosis was discussed (*n* = 65, as per the physician's response), the majority were those who had a prognosis of ≥1 year (*n* = 45; 69.2%) as opposed to patients who were given a prognosis of <1 year (*n* = 20; 30.8%).

As the understanding of treatment intent and prognosis had the highest discordant rates between patients and physicians, we conducted separate univariable logistic regression analyses to determine which factors were associated with concordance rates of treatment intent and prognosis (Table 3). In regard to treatment intent, we did not find any statistically significant associations. Our analysis of prognostic concordance, however, found male patients (OR = 3.03; 95% CI: 1.61–5.72) to be more likely in agreement with their oncologist. Conversely, the duration of practice as an attending medical oncologist (OR = 0.95; 95% CI: 0.92–0.98) was inversely associated with prognostic concordance.

**TABLE 3:** Univariable logistic regression analyses of patient–physician concordance on treatment intent and prognosis as outcomes.

Covariate	Univariable analysis (Outcome = Treatment Intent)			Univariable analysis (Outcome = Prognosis)		
	OR (95% CI)	*p*‐value	*n*	OR (95% CI)	*p*‐value	*n*
Age	1.01 (0.99–1.04)	0.40	178	1.00 (0.97–1.02)	0.86	175
Sex, male	1.00 (0.53–1.89)	0.99	178	**3.03 (1.61–5.72)**	**<0.001**	175
Prior history of cancer	0.87 (0.40–1.89)	0.72	178	0.91 (0.54–1.94)	0.81	175
Education, high school, or below	1.60 (0.75–3.43)	0.22	178	0.96 (0.48–1.93)	0.91	175
Primary language other than English	0.69 (0.32–1.51)	0.36	174	0.90 (0.41–1.95)	0.79	171
Patient did not bring questionnaire home to complete	0.52 (0.24–1.13)	0.10	174	0.99 (0.49–1.99)	0.98	175
Family member was present at consultation	0.76 (0.36–1.62)	0.48	178	0.87 (0.43–1.76)	0.70	175
Patient was seen by an external medical oncologist prior to medical oncology consultation at PMH	1.65 (0.70–3.92)	0.25	178	0.74 (0.35–1.57)	0.43	175
Patient was seen by a surgical and/or radiation oncologist prior to medical oncology consultation at PMH	0.97 (0.49–1.91)	0.94	178	0.51 (0.26–1.01)	0.052	175
Patient first reviewed by medical trainee before staff medical oncologist	0.65 (0.29–1.45)	0.29	177	0.81 (0.38,1.71)	0.58	175
Years of experience as a staff medical oncologist	1.01 (0.98–1.04)	0.68	178	**0.95 (0.92–0.98)**	**<0.001**	175

### 3.2 Patient satisfaction

**FIGURE 1:**
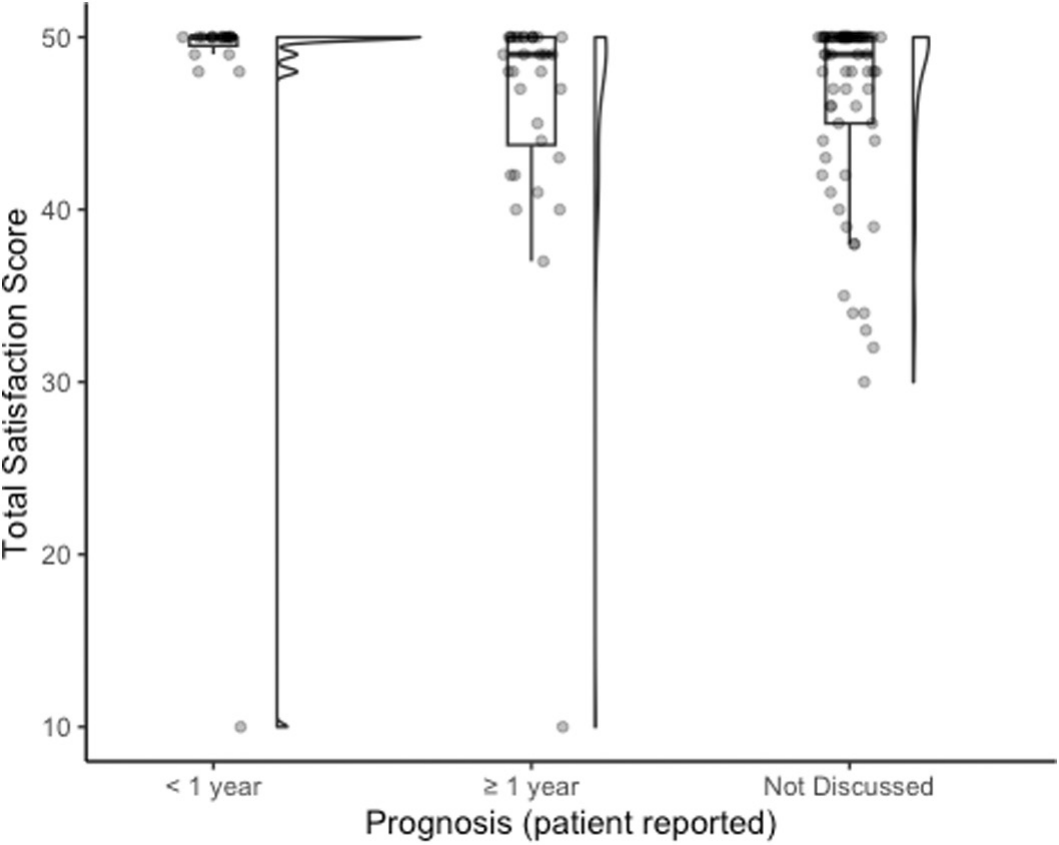
Patient satisfaction of communication stratified by patient's perception of prognosis (*p* = 0.043).

The mean scores for each satisfaction item can be found in Table 4. Based on the 10 items (Data S1) included in the satisfaction section of the questionnaire, the two areas that showed the lowest satisfaction scores (i.e., 0 = strongly disagree, 1 = disagree, or 2 = neither agree/disagree) were communication on (1) prognosis (12.5%) and (2) the way that their physician explored the impact of their diagnosis on their life (15.2%). Patients were most satisfied (i.e., 4 = agree or 5 = strongly agree) with the way their doctor answered their questions (95.7%) and 95.1% of patients felt assured that their doctor had their best interests in mind. They were also satisfied with how they were included in the decisions made around their care (94.5%). Overall, satisfaction with the quality of communication that patients received during their first medical oncology visit was high (total mean score out of 50 = 46.5 ± 6.5). Internal reliability of the satisfaction questionnaire was high (Cronbach's *α* = 0.947). In Figure 1, total satisfaction scores were stratified based on patient‐reported prognosis which showed statistically significant differences between the subgroups (prognosis <1 year; prognosis ≥1 year; prognosis not discussed) (*p* = 0.043). We did not find any statistically significant differences between patient‐reported treatment intent subgroups (results not shown).

**TABLE 4:** Patient satisfaction with communication at initial medical oncology consultation. Each item was scored on a 5‐point Likert scale.

Satisfaction item	Mean (SD)	*n*
1) My doctor communicated my diagnosis to me clearly and in a way I understand.	4.75 (0.64)	178
2) My doctor communicated the recommended treatment plan in a way I understand.	4.70 (0.68)	173
3) My doctor communicated all treatment options in a way I understand.	4.64 (0.81)	173
4) My doctor communicated treatment intention (curative vs palliative) in a way I understand.	4.62 (0.81)	161
5) My doctor communicated my prognosis to me in a way I understand.	4.45 (0.98)	143
6) My doctor answered all my questions, to the best of their ability.	4.80 (0.64)	183
7) I appreciate the way my doctor tried to explore the impact of my diagnosis on my life.	4.42 (0.97)	161
8) I feel comfortable that my doctor is going to make every effort to help me during this time.	4.76 (0.66)	182
9) I feel confident that my doctor has my best interests in mind.	4.80 (0.64)	182
10) I feel included in the decisions being made around my care moving forward.	4.69 (0.70)	179
Total score^a^	46.47 (6.55)	127

^a^ Calculated by adding each item for patients who rated all 10 items.

Uni‐ and multivariable analyses were conducted to assess potential factors associated with patient satisfaction on patient–physician communication. As shown in Table 5, patients completing their questionnaire immediately after their consultation were significantly more likely to give a higher total satisfaction score than those who took their questionnaire home (adjusted estimate = 2.76; 95% CI: 0.72–4.81). However, total patient satisfaction scores were significantly lower if patients reported that the Intent of their treatment was unclear at time of consultation (adjusted estimate = −3.32; 95% CI: ‐5.93 ‐ ‐0.70). There was no statistical significance between patient‐reported prognosis and their satisfaction in communication.

**TABLE 5:** Uni/multivariable linear regression analysis of total patient satisfaction scores.

Covariate	Univariable analysis		Multivariable analysis		
	Estimates (95% CI)	*p*‐value	Estimates (95% CI)	*p*‐value	*n*
Age	0.01 (−0.08 to 0.11)	0.8	0.08 (−0.006 to 0.16)	0.066	127
Sex, male	2.50 (0.21 to 4.79)	0.03	1.02 (−0.69 to 2.73)	0.24	127
Prior history of cancer	−1.48 (−4.38 to 1.41)	0.31	−0.62 (−2.98 to 1.75)	0.60	126
Education (college or higher as reference)		0.33		0.34	127
High school or below	0.85 (−1.74 to 3.43)	0.52	1.48 (−0.67 to 3.64)	0.18	
Other/missing	−2.37 (−6.38 to 1.65)	0.25	1.33 (−1.87 to 4.54)	0.41	
Primary language other than English	−0.97 (−2.99 to 1.05)	0.34	−1.98 (−4.31 to 0.34)	0.09	124
Patient questionnaire completed immediately after consultation	1.40 (−1.17 to 3.97)	0.28	2.76 (0.72 to 4.81)	0.0074	127
Family member was present at consultation	−1.00 (−3.58 to 1.57)	0.44	−0.05 (−1.97 to 1.87)	0.96	127
Patient perception on treatment intent		0.058		0.042	124
Palliative	−0.57 (−3.10 to 1.96)	0.66	−1.15 (−3.23 to 0.94)	0.28	
Unclear	−3.92 (−7.25 to −0.59)	0.021	−3.32 (−5.93 to −0.70)	0.014	
Prognosis not discussed^a^	−0.01 (−2.42 to 2.40)	0.99	−1.58 (−3.38 to 0.21)	0.08	122
Patient was seen by an external medical oncologist prior to medical oncology consultation at PMH^b^	−0.54 (−3.44 to 2.36)	0.71	‐	‐	127
Patient was seen by a surgical and/or radiation oncologist prior to medical oncology consultation at PMH	0.15 (−2.29 to 2.59)	0.9	0.36 (−1.59 to 2.32)	0.71	127
Patient first reviewed by medical trainee before staff medical oncologist	−2.09 (−4.98 to 0.80)	0.15	−1.21 (−3.34 to 0.91)	0.26	126
Years of practice of treating medical oncologist	−0.01 (−0.13 to 0.10)	0.81	0.0048 (−0.08 to 0.09)	0.91	127

^a^ Defined as “doctor did not address” or “patient requested not to discuss” based on patient responses.

^b^ Cannot be considered in the multivariable model since all records were labeled as “No” when considering non‐missing cases.

## 4 DISCUSSION

A heavy emphasis has been placed on patient‐centered care and informed decision‐making across all medical specialties in the last few decades. Not only was this study an internal quality assurance check in a single center, we objectively measured patient understanding and satisfaction of the information delivered specifically to patients with a GI malignancy at the initial medical oncology consultation. Patient–physician concordance was highest in cancer diagnosis (92.9%). Furthermore, all patients were able to select at least one treatment modality that was concordant with the physicians' response (59.2% fully concordant; 40.8% partially concordant). However, lower concordance rates were observed in patients' understanding of the intent of their treatment (66.8%) and their prognosis (59.8%). Patient satisfaction was generally high, although there were a few factors that were independently associated with lower satisfaction scores.

### 4.1 Patient understanding of treatment intent

The terms “curative” versus “palliative” intent in the context of cancer treatment are often misunderstood by patients, caregivers, and even among health‐care providers.^35^, ^36^ While active anticancer treatment in the palliative (i.e., non‐curative) setting is given to control the progression of cancer, optimize symptom control, and hopefully, prolong survival, many patients perceive any systemic chemotherapy and/or radiation as a means to cure their cancer. On the other hand, patients may misinterpret “palliative intent” as being ineligible for any type of anticancer treatment and may cause additional distress.

In this study, 123 (66.8%) patients selected the same answer as their oncologist for treatment intent (curative vs palliative). Over 75% of discordant cases were patients who believed their cancer to be curable or that more tests were necessary to establish a treatment plan, but in fact, were deemed incurable by their medical oncologist. Concordance rates on the understanding of treatment goals in Western and non‐Western countries range from 29% to 76%, suggesting that the challenges physicians face in communicating treatment intent are universal.^2^, ^3^, ^4^, ^5^, ^6^, ^7^

Focusing on the distinction between “curative” versus “palliative” intent and the role of palliative care services in the context of cancer is important early on, as it allows future conversations with patients to be based on an accurate understanding of the purpose of their treatment.

### 4.2 Patient understanding of prognosis

There is significant variability in the attitudes and practices of oncologists giving “bad news” to patients. Although geographical, cultural, and familial factors may influence the disclosure of unfavorable medical information,^37^, ^38^, ^39^ there is also an inherent hesitation from physicians, who, understandably, worry about the emotional well‐being and relationship with their patients. Not surprisingly, the lowest concordance rate in this study was cancer prognosis (59.8%) (Table 2). Based on physician responses, prognosis was not discussed during the initial consultation in 113 (62%) of the cases, in addition to the five (3%) participants who specifically requested not to discuss prognosis. Interestingly, nearly 70% of patients with which prognosis was discussed (physician‐reported) were given a prognosis of ≥1 year, whereas the remaining patients had <1 year, suggesting that oncologists may be subconsciously more likely to disclose prognosis if it was favorable.

We did not perform a multivariable logistic regression analysis in patient–physician concordance rates as nearly 75% of our patients were assisted by their caregivers in the completion of the research questionnaire and hence, not an accurate depiction of how patient factors (e.g., education level, primary language) influence concordance. Weeks et al., found that the risk of reporting inaccurate beliefs about chemotherapy were dependent on cancer site (lung vs. colorectal), ethnicity, and patients' satisfaction with physician communication—regardless of education level, functional status, and patients' role in the decision‐making process.^4^ In our univariable logistic regression analysis, we showed that the odds of achieving patient‐physician concordance on prognosis was significantly higher if the patient was seen by a physician with less years of medical oncology experience (Table 3). This may reflect the impact of an evolving medical curriculum that emphasizes patient‐centered care and strategies for high‐quality goals of care conversations for trainees in recent decades.

### 4.3 Patient satisfaction on physician–patient communication

This study's secondary objective was to assess patients' satisfaction with the communication with their physician based on the *first* medical oncology consultation as it sets the tone throughout a patient's cancer journey.

In our multivariable linear regression analysis, independent predictors of lower *overall* satisfaction in our patients included: (1) those who completed the questionnaire at home rather than immediately after the initial consultation; and (2) those who reported that treatment intent was unclear at time of the consultation (i.e., more diagnostic tests were needed) (Table 5). However, what was more insightful was the absence of statistical significance for patient‐reported prognosis. Compared to patients who reported that prognosis was disclosed, patients were neither less nor more satisfied if prognosis was not disclosed (i.e., physician did not address it or patient requested not to discuss it). Although prognosis should be given at the patient's request, physician hesitancy to address the topic due to concerns of patient well‐being and their relationship with the patient should be alleviated, as we and many other studies showed that discussing prognosis was not associated with worse patient–physician relationship ratings, sadness, or anxiety.^2^, ^3^, ^8^, ^9^, ^10^, ^11^, ^12^

### 4.4 Implications for practice

This study revealed present challenges that medical oncologists encounter when delivering important and sensitive information to patients during a consultation. The application to the current practice of cancer medicine is evident—patient understanding of treatment intent and prognosis were areas that need the most improvement—similar to what has been previously published.^2^, ^3^, ^4^, ^5^, ^6^, ^7^, ^8^, ^9^, ^10^, ^11^, ^12^, ^13^, ^14^, ^15^, ^16^ Small but impactful changes such as focusing the discussion on defining key phrases like “curative vs. palliative treatment intent” and “median survival” can be made relatively quickly. In terms of patients who reported lower satisfaction scores if treatment intent was unclear at the time of the consultation, perhaps holding multidisciplinary cancer conferences *prior* to the consultation may help the clinical team agree on a general treatment plan/intent and avoid giving contradictory information to their patients.

Physician‐targeted interventions such as *OncoTalk* designed by Back et al.^18^ may be considered to strengthen the communication between oncologists and patients. This experimental curriculum demonstrated that the 4‐day workshop significantly improved skills in “giving bad news.” Other aids that are less time‐demanding such as the SPIKES protocol developed by Baile et al.^24^ have been found to improve learner satisfaction, knowledge, and performance.^40^ However, patient outcomes following physician‐targeted interventions are not well studied and warrants further investigation.

Consultation aids developed for patients may also be considered. Previous studies have found that patients asked significantly more questions about prognosis if they were provided a question prompt list than those with a general fact sheet prior to the consultation.^27^, ^28^, ^29^, ^30^, ^31^ However, they were also significantly more anxious than the control group and were less likely to achieve their preferred decision‐making style. This was alleviated if the aids were endorsed by the oncologist during the consultation,^28^, ^31^ indicating that both patients' and physicians' behavior must be targeted to improve the experience. Real‐world implementation of patient‐ and physician‐based interventions will depend on patient‐, provider‐, and system‐level barriers.

### 4.5 Limitations

There were some limitations to this study. The first limitation was selection bias as non‐English speaking patients who were not accompanied by an English‐speaking caregiver were not approached. Thus, the concordance rates may be overestimated. Along these lines, as we did not provide questionnaires in languages other than English, participants who speak English as a second language may have had issues fully understanding certain questions. This was also a single‐center study taking place in the GI oncology clinic at an academic hospital where patients would see a medical trainee prior to the staff oncologist and thus, may not be generalizable to other disease sites nor community‐based centers.

Secondly, recall bias from the patient as well as the physician may affect concordance rates if the questionnaire was completed immediately or a few days after the consultation. The direction of which this affects concordance is unknown as completing the patient questionnaire at home may allow the patient to talk to family and friends and research their illness through other resources, but it can also reduce the accuracy of recalling information discussed with their medical oncologist. Of note, total patient satisfaction scores were significantly higher when patients completed the questionnaire in clinic compared to completing it at home, which suggests that patients may have felt more inclined to give a higher rating when their oncologist was in closer proximity.

Lastly, this study did not address whether patients' level of understanding of treatment intent and prognosis improved over consecutive visits. This may not be something we can “perfect” in the first visit, given the emotional toll of being told one has cancer and we acknowledge that as the patient–physician relationship evolves, the concordance in understanding evolves as well.

## 5 CONCLUSION

This study was one that focused specifically on GI malignancies and the only one to assess patient understanding and satisfaction after the *first* consultation with their medical oncologist. It objectively assessed a more homogeneous group of specialists (i.e., medical oncologists) at communicating complex ideas with patients during and identified areas for improvement in our clinic. We observed that concordance in the understanding of treatment intent and prognostic disclosure were suboptimal. It is reasonable to have high‐quality conversations regarding goals of care and prognosis early on in the oncologist–patient relationship.

## AUTHOR CONTRIBUTIONS

**Yvonne Bach:** Data curation (lead); formal analysis (supporting); investigation (equal); methodology (supporting); project administration (equal); writing – original draft (lead); writing – review and editing (lead). **Elan Panov:** Conceptualization (lead); data curation (equal); formal analysis (supporting); investigation (equal); methodology (lead); project administration (equal); writing – review and editing (lead). **Osvaldo Espin‐Garcia:** Formal analysis (lead); writing – review and editing (supporting). **Eric Chen:** Project administration (equal); resources (equal); supervision (equal); writing – review and editing (equal). **Monika Krzyzanowska:** Project administration (equal); resources (equal); supervision (equal); writing – review and editing (equal). **Grainne O'Kane:** Project administration (equal); resources (equal); supervision (equal); writing – review and editing (equal). **Malcolm Moore:** Project administration (equal); resources (equal); supervision (equal); writing – review and editing (supporting). **Rebecca M. Prince:** Project administration (equal); resources (equal); supervision (equal); writing – review and editing (supporting). **Jennifer Knox:** Project administration (equal); resources (equal); supervision (equal); writing – review and editing (supporting). **Robert Grant:** Project administration (equal); resources (equal); supervision (equal); writing – review and editing (supporting). **Lucy X. Ma:** Project administration (supporting); writing – review and editing (equal). **Michael J. Allen:** Project administration (supporting); writing – review and editing (equal). **Lawson Eng:** Project administration (supporting); writing – review and editing (supporting). **Ekaterina Kosyachkova:** Project administration (supporting); writing – review and editing (equal). **Thais Baccili Cury Megid:** Writing – review and editing (equal). **Carly Barron:** Writing – review and editing (equal). **Xin Wang:** Writing – review and editing (equal). **Marie‐Philippe Saltiel:** Writing – review and editing (equal). **Abdul Rehman Rehman Farooq:** Writing – review and editing (equal). **Raymond W. Jang:** Conceptualization (equal); methodology (equal); project administration (equal); resources (equal); supervision (lead); writing – review and editing (equal). **Elena Elimova:** Conceptualization (equal); funding acquisition (lead); investigation (lead); methodology (equal); project administration (equal); resources (lead); supervision (lead); writing – review and editing (equal).

## FUNDING INFORMATION

This study was funded by a generous donation through the Princess Margaret Cancer Foundation.

## CONFLICT OF INTEREST STATEMENT

There are no disclosures to report.

## ETHICS STATEMENT

This study received ethics approval from the University Health Network Review Ethics Board (CAPCR ID: 20‐6119).

## Supporting information


Data S1.

Data S2.

## Data Availability

The data that support the findings of this study are available on request from the corresponding author. The data are not publicly available due to privacy or ethical restrictions.
