# Supplementary material for: First impressions: A prospective evaluation of patient–physician concordance and satisfaction following the initial medical oncology consultation
Source: Cancer Med. 2023 Dec 8;12(24):22293–303. doi: 10.1002/cam4.6758 (PMC10757128; doi:10.1002/cam4.6758)
Supplement: Supplementary file 1 — Data S1. [file CAM4-12-22293-s002.pdf]

The following questionnaire is part of a study titled

First impressions: Are oncologists effective in conveying their message to patients during the initial clinical encounter?

PATIENT STUDY ID: ________
DATE COMPLETED: ________

INSTRUCTIONS

1. For each question, please place a checkmark (√) in the circle next to your preferred answer.
2. If you agree to fill out this questionnaire, we ask that you do so prior to your next visit.
3. When finished, place the entire questionnaire in the envelope provided and return it on your subsequent visit with your oncologist.
    - You may fill out the questionnaire now and leave it with us.
    - You may take it home and bring it with you to your next appointment.
    - If you do not have a follow up appointment, you may mail in your answers using the pre-paid envelope provided.

1. **Demographics**

Age	_________		
Sex	○ Female	○ Male	○ Other
Education	○ No formal education	○ Grade school	○ High school
	○ College/University	○ Doctorate/PhD	○ Other ________
Primary language	○ English	○ Other ________	

2. **How was this consultation performed?**

○ In person ○ Video conference (OTN) ○ Phone

3. **Have you previously seen another specialist – Surgeon, Radiation oncologist, Medical oncologist – for your diagnosis? If so, was that meeting in person, by video conference, or by phone?**

○ YES – In person

○ YES – By phone

○ NO, I have not seen another specialist for my diagnosis

4. **Was a family member or friend present with you during the initial consultation? If so, were they present in person or by phone?**

○ YES – In person

○ YES – By phone

○ NO, a family member was not present

5. **What type of cancer have you been diagnosed with?**

○ Gastroesophageal (such as Esophagus, GE junction, Stomach)

○ Bowel (such as Small bowel, Colon, Rectum, Anus)

○ Hepatobiliary (such as Liver, Pancreas, Cholangiocarcinoma/Bile duct, Ampullary)

○ Neuroendocrine (“NET”)

○ Type of cancer is unclear right now, more investigations needed

6. What treatment(s) does your doctor propose at this time?
(CHECK ALL THAT APPLY)

- ○ Drug therapy (such as oral or intravenous medication)
- ○ Surgery
- ○ Radiation therapy
- ○ "Best Supportive Care" (symptom management only)
- ○ Treatment unclear

7. What is the purpose of the above treatment?

- ○ Curative (eliminate cancer and achieve cure)
- ○ Palliative (control cancer, improve symptoms – cure is not possible)
- ○ Unclear at this time, pending further investigations and/or referrals

8. Was prognosis/survival outcomes discussed? If so, do you recall what was said?

- ○ YES, I was told "Months" or "Less than 1 year"
- ○ YES, I was told "1 year," "More than 1 year" or "Years"
- ○ NO, my doctor did not address prognosis/survival time
- ○ NO, I requested not to discuss prognosis/survival time

9. What aspects of the conversation do you feel your doctor emphasized most?
(CHECK ALL THAT APPLY)

- ○ Diagnosis
- ○ Further testing
- ○ Treatment
- ○ Purpose of treatment
- ○ Prognosis
- ○ Impact on your life

10. Are there any other comments you wish to share?

____

____

To what extent do you agree or disagree with the following statements?
For each statement, please circle one number, which corresponds to your level of agreement

1 = Strongly disagree	2 = Disagree	3 = Neither agree/disagree	4 = Agree	5 = Strongly agree

1. My doctor communicated my diagnosis to me clearly and in a way I understand.

   1 2 3 4 5 N/A

2. My doctor communicated the recommended treatment plan in a way I understand.

   1 2 3 4 5 N/A

3. My doctor communicated all treatment options in a way I understand.

   1 2 3 4 5 N/A

4. My doctor communicated treatment intention (curative vs palliative) in a way I understand.

   1 2 3 4 5 N/A

5. My doctor communicated my prognosis to me in a way I understand.

   1 2 3 4 5 N/A

6. My doctor answered all of my questions, to the best of their ability.

   1 2 3 4 5 N/A

7. I appreciate the way my doctor tried to explore the impact of my diagnosis on my life.

   1 2 3 4 5 N/A

8. I feel comfortable that my doctor is going to make every effort to help me during this time.

   1 2 3 4 5 N/A

9. I feel confident that my doctor has my best interests in mind.

   1 2 3 4 5 N/A

10. I feel included in the decisions being made around my care moving forward.

    1 2 3 4 5 N/A

The following page can be used to write down any questions or concerns you may have following your first visit. If there are any outside resources you have turned to (such as internet sites, articles, family, friends), please write them in the spaces below as well. Completing this page, in addition to the others, will give doctors a better understanding into how patients make sense of the information they are given. However, you may choose to leave this section blank, if preferred.

1. **Do you have a follow up appointment scheduled with your Oncologist?**

   ○ YES

   ○ NO

2. **In the space below, please write down any questions or concerns you have following the initial visit with your medical oncologist (such as specific questions about your diagnosis, tests, treatment, prognosis/survival).**

   ____

   ____

   ____

3. **In the space below, please list any resources you may have turned to (such as websites, articles, books) for more information about your disease following your initial consult visit at Princess Margaret Cancer Centre.**

   ____

   ____

   ____
