# Supplementary material for: First impressions: A prospective evaluation of patient–physician concordance and satisfaction following the initial medical oncology consultation
Source: Cancer Med. 2023 Dec 8;12(24):22293–303. doi: 10.1002/cam4.6758 (PMC10757128; doi:10.1002/cam4.6758)
Supplement: Supplementary file 2 — Data S2. [file CAM4-12-22293-s001.pdf]

The following questionnaire is part of a study titled

First impressions: Are oncologists effective in conveying their message to patients during the initial clinical encounter?

PATIENT STUDY ID: _________
DATE COMPLETED: _________

Instructions

1. For each question, please place a checkmark (**√**) in the circle next to your preferred answer.
2. If you agree to fill out this questionnaire, we ask that you do so within 24hrs of the consult.
3. When finished, put the entire questionnaire in the envelope provided and place it in the bin at the clinic reception.
4. If you do not wish to fill out this questionnaire, please leave it blank, place it in the envelope provided, and put it in the bin at the clinic reception regardless.

1. **Demographics**

   Years in practice ○ < 5 ○ 5-10 ○ 11-20 ○ 20 +

2. **How was this consultation performed?**

   ○ In person ○ Video conference (OTN) ○ Phone

3. **Did a resident or fellow perform the consultation with you?**

   ○ YES ○ NO

4. **Was a family member or friend present during the initial consultation?**

   ○ YES – In person

   ○ YES – By phone

   ○ NO, a family member did not attend the consultation

5. **What type of cancer is the patient diagnosed with?**

   ○ Gastroesophageal (Esophagus, GE junction, Stomach)

   ○ Bowel (Small bowel, Colon, Rectum, Anus)

   ○ Hepatobiliary (HCC, Pancreas, Cholangiocarcinoma, Ampullary)

   ○ Neuroendocrine

   ○ Type of cancer is unclear right now

6. **What treatment (if any) was proposed at this time? CHECK ALL THAT APPLY**

   ○ Systemic therapy

   ○ Surgery

   ○ Radiation therapy

   ○ Best Supportive Care

   ○ Treatment unclear (need further tests, referrals, discussion at Tumor Board Rounds)

7. What is the purpose of the above treatment?

- ○ Curative
- ○ Palliative
- ○ Unclear at this time, pending further investigations and/or referrals

8. Was prognosis with proposed treatment discussed? If so, what was it?

- ○ YES, “Months” or “Less than 1 year”
- ○ YES, “1 year,” “More than 1 year” or “Years”
- ○ NO, I did not address prognosis
- ○ NO, the patient requested not to discuss prognosis

9. What aspect of the consultation do you feel the patient did NOT fully understand? (CHECK ALL THAT APPLY)

○ Diagnosis	○ Further testing	○ Treatment
○ Purpose of treatment	○ Prognosis	○ Impact on their life

10. Are there any other aspects of the consultation (i.e. patient comprehension, patient attitude, personal attitude) you wish to highlight or comment on?

____

____

____

____
